# Osteoarthritis of the Temporomandibular Joint can be diagnosed earlier using biomarkers and machine learning

**DOI:** 10.1038/s41598-020-64942-0

**Published:** 2020-05-15

**Authors:** Jonas Bianchi, Antônio Carlos de Oliveira Ruellas, João Roberto Gonçalves, Beatriz Paniagua, Juan Carlos Prieto, Martin Styner, Tengfei Li, Hongtu Zhu, James Sugai, William Giannobile, Erika Benavides, Fabiana Soki, Marilia Yatabe, Lawrence Ashman, David Walker, Reza Soroushmehr, Kayvan Najarian, Lucia Helena Soares Cevidanes

**Affiliations:** 1 University of Michigan, Department of Orthodontics and Pediatric Dentistry, School of Dentistry, Ann Arbor, MI 48109 USA; 2 São Paulo State University (UNESP), Department of Pediatric Dentistry, School of Dentistry, Araraquara, SP 14801-385 Brazil; 3 Kitware, Inc., Carrboro, NC 27510 USA; 4 University of North Carolina, Department of Psychiatry and Computer Science, Chapel Hill, NC 27516 USA; 5 University of North Carolina, Department of Biostatistics, Chapel Hill, NC 27516 USA; 6 University of Michigan, Department of Periodontics and Oral Medicine, School of Dentistry, Ann Arbor, MI 48109 USA; 7 University of Michigan, Department of Oral and Maxillofacial Surgery and Hospital Dentistry, School of Dentistry, Ann Arbor, MI 48109 USA; 8 University of North Carolina, Department of Orthodontics, Chapel Hill, NC 27516 USA; 9 University of Michigan, Center for Integrative Research in Critical Care and Michigan Institute for Data Science, Department of Computational Medicine and Bioinformatics, Ann Arbor, MI 48109 USA

**Keywords:** Three-dimensional imaging, Diagnosis, Dentistry, Orthodontics

## Abstract

After chronic low back pain, Temporomandibular Joint (TMJ) disorders are the second most common musculoskeletal condition affecting 5 to 12% of the population, with an annual health cost estimated at $4 billion. Chronic disability in TMJ osteoarthritis (OA) increases with aging, and the main goal is to diagnosis before morphological degeneration occurs. Here, we address this challenge using advanced data science to capture, process and analyze 52 clinical, biological and high-resolution CBCT (radiomics) markers from TMJ OA patients and controls. We tested the diagnostic performance of four machine learning models: Logistic Regression, Random Forest, LightGBM, XGBoost. Headaches, Range of mouth opening without pain, Energy, Haralick Correlation, Entropy and interactions of TGF-β1 in Saliva and Headaches, VE-cadherin in Serum and Angiogenin in Saliva, VE-cadherin in Saliva and Headaches, PA1 in Saliva and Headaches, PA1 in Saliva and Range of mouth opening without pain; Gender and Muscle Soreness; Short Run Low Grey Level Emphasis and Headaches, Inverse Difference Moment and Trabecular Separation accurately diagnose early stages of this clinical condition. Our results show the XGBoost + LightGBM model with these features and interactions achieves the accuracy of 0.823, AUC 0.870, and F1-score 0.823 to diagnose the TMJ OA status. Thus, we expect to boost future studies into osteoarthritis patient-specific therapeutic interventions, and thereby improve the health of articular joints.

## Introduction

Osteoarthritis (OA) affects millions of people worldwide, causing them many years with pain and disability^1^. Trends in the global burden of the disease from 1990 to 2016 show that OA is the second most rapidly rising condition associated with disability, with a 46 percent increase in years lived with disability, just behind diabetes at 52 percent^2^. With the aging population and higher rates of obesity, this burden is expected to rise. The rapid increase in the prevalence of OA will lead to a growing impact and major challenges for health care and public health systems. OA can occur in different joints in the musculoskeletal system, such as the knee, hips, back, hand, and temporomandibular joint (TMJ), having a multifactorial etiology that includes: excessive mechanical stress, hormonal changes, genetics, aging and others^3–5^. The TMJ is a unique model to study early bone changes in OA, as the articular bone surface is covered only by a thin layer of fibrocartilage in the TMJ condyle. Osteoarthritis of the TMJ (TMJ OA) is a multi-system disease, involving numerous pathophysiological processes, and requiring comprehensive assessments to characterize progressive cartilage degradation, subchondral bone remodeling, and chronic pain^4,6–8^.

Studies using *in vivo* OA disease models now benefit from high-resolution cone-beam tomography imaging (HR-CBCT)^9^. HR-CBCT scans allow diagnosis of the bone environment with sub-millimeter resolution comparable to micro-CT, but with a much lower radiation dose^10^, and have been widely used by clinicians and researchers^11–14^. As treatments to reverse the chronic damage of TMJ OA are unavailable, early diagnosis may provide the best opportunity to prevent extensive and permanent joint damage. However, current diagnosis is based on pre-existent clinical/imaging signs and symptoms markers using standard protocols recommended for Diagnostic Criteria for Temporomandibular Disorders (DC/TMD), meaning to diagnose TMJ OA degradation of the joint must have already occurred^15,16^. The DC/TMD criteria are based on pre-existent condylar damage, such as subcortical cysts, surface erosions, osteophytes, or generalized sclerosis that are present mainly in the later stages of the disease. Towards an early diagnosis that is predictive of disease status, animal studies indicate that the bone microarchitecture^6,8,17,18^ is an important factor in the OA pathogenesis initiation, preceding articular cartilage changes^17,19^, and should be investigated in human studies. There is an estimated increase in OA prevalence over the next decades, which reflects in more data acquisition, demanding advances in computational machine learning and data management^20–22^. For this reason, there is a need for precise data mining algorithms, data capture, standardization, management and processing from multiple centers to provide personalized treatment and diagnosis^15,20,22–25^. For disease diagnosis, machine learning approaches have been applied in the medical field^26–29^. Most of the studies have pointed out algorithms and multi-source biomarkers to predict the disease status, such as XGBoost^30^, LightGBM^31^, deep learning algorithms^32^, random forest algorithms^27^, etc. The models have been tested with different features, including radiographic and magnetic resonance (MRI) data^33,34^, proteomics^28^, and clinical information^27^ for creating patient-specific prediction models^23^. However, most studies addressed the OA involvement in the knee. For the temporomandibular joint, we found two studies that were done by our group evaluating only the morphological changes in mandibular condyles^35,36^. In addition, most of the literature is focused on multi-center database, or late stages of OA (chronic stages) assessed using routine exams. Here, we addressed surrogate biomarkers such as the radiomics, which can be useful to explore the subchondral bone organization and maybe play a pivotal role in a true early diagnosis of the TMJ OA.

**Figure 1:**
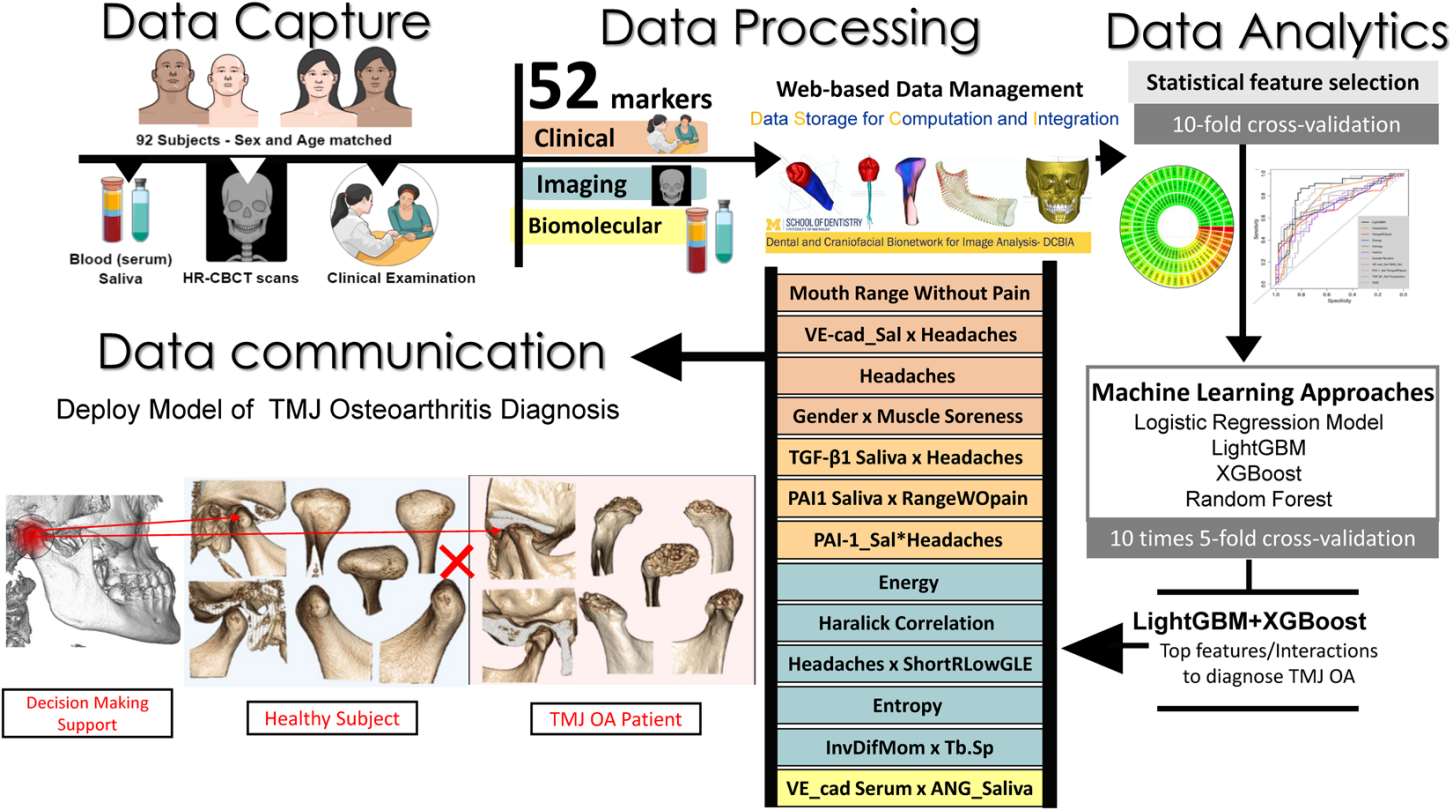
The spectrum of Data Science to advance TMJ OA diagnosis includes Data capture and acquisition, Data processing with a web-based data management, Data Analytics involving in-depth statistical analysis, machine learning approaches, and Data communication to help the decision-making support in TMJ OA diagnosis.

We propose novel standardized data representation/processing, statistical learning, and interactive visualization to fully explore biomarker interactions to disease and health. Our data-driven approaches integrate information patterns to provide new insights into the complex etiology of TMJ OA^37^. Data management includes standardized imaging^38^, clinical^15^ and biomolecular^39^ acquisition, and control of patient information from multiple data sources, with standardized demographic for matching OA patients and healthy controls. We have evaluated fifty-two variables to determine the most relevant integrative feature pools using machine-learning algorithms to detect TMJ OA status (Fig. 1). We hypothesize that by combining standardized patient features from multiple sources using statistical machine-learning approaches, we can accurately diagnose TMJ OA status.

## Results

### Web-based platform to store and compute data analytics of clinical, radiomics and biomolecular markers

Our Data Storage for Computation and Integration (DSCI)^40^ web-based system was used for data management with storage and integration of patient information from multiple sources. The DCSI communicates with 3D Slicer^41^ platform through the Data Base lnteractor^42^ plugin that allows the user to upload the clinical, imaging and biological markers. The data was exported in a.csv file and we show in Tables 1–3, the descriptive statistics for each variable and their respective nomenclature. As most of the variables did not show parametric distribution (after evaluation of the asymmetry, kurtosis and Shapiro-wilk test), the descriptive statistics display the median, mean, standard deviation and upper/lower limits for the 95% confidence interval. The following variables were measured only for the TMJ OA group since the control patients did not present facial and/or TMJ pain: years of pain onset (PainY), current facial pain (PainCur), last six months worst facial pain (PainWor) and last six months average facial pain (PainAve). The TMJ OA and control groups were age and sex matched. We can note that patients with OA present less range of mouth opening, for radiomics and biomolecular variables both present similar values and in Supplementary Fig. 1 we present the statistical differences between them. Finally, the MMP3 protein was not described for saliva, since the expression levels were not detected.

**Table 1:** Descriptive and demographic values for each clinical variable.

Variables	Abbreviation	Control Group (n = 46) Female (39) Male (7)					TMJ OA Group (n = 46) Female (39) Male (7)				
		Median	Mean	95% CI		SD	Median	Mean	95% CI		SD
Clinical Variables				Lower	Upper				Lower	Upper	
Age	Age	38.50	39.83	35.89	43.76	13.26	38.00	37.65	33.99	41.32	12.34
Years of Pain Onset (years)	PainY	—	—	—	—	—	3.75	4.34	3.35	5.33	3.34
Facial Current Pain (years)	PainCur	—	—	—	—	—	3.00	3.07	2.47	3.66	2.00
Facial last 6 months Worst Pain (0 to 10)	PainWor	—	—	—	—	—	7.00	6.89	6.06	7.73	2.81
Facial last 6 months Average Pain (0 to 10)	PainAve	—	—	—	—	—	4.50	4.52	3.87	5.17	2.20
Last 6 Months Distressed by Headaches (0 to 10)	Headaches	0.00	0.63	0.33	0.93	1.02	2.00	1.65	1.33	1.97	1.08
Last 6 Months Distressed by Muscle Soreness (0 to 10)	MusSor	0.00	0.37	0.16	0.58	0.71	1.00	1.07	0.74	1.39	1.10
Vertical Range Unassisted Without Pain (mm)	RangeWOpain	44.50	44.91	42.42	47.40	8.39	36.35	39.00	32.44	40.26	13.16
Vertical Range Unassisted Max (mm)	RangeUnaMax	47.50	46.83	44.62	49.03	7.41	45.00	44.28	41.41	47.16	9.68
Vertical Range Assisted Max (mm)	RangeAssMax	50.00	49.21	47.15	51.27	6.94	49.00	47.54	44.69	50.40	9.61

CI: Confidence Interval; SD: Standard Deviation.

**Table 2:** Descriptive values for each imaging variable.

Variables	Abbreviation	Control Group (n = 46) Female (39) Male (7)					TMJ OA Group (n = 46) Female (39) Male (7)				
		Median	Mean	95% CI		SD	Median	Mean	95% CI		SD
Radiomics Variables				Lower	Upper				Lower	Upper	
Energy	Energy	0.30	0.31	0.28	0.33	0.07	0.25	0.25	0.23	0.27	0.07
Entropy	Entropy	2.29	2.30	2.20	2.40	0.33	2.56	2.62	2.49	2.75	0.42
Inverse Difference Moment	InvDifMom	0.91	0.90	0.90	0.91	0.02	0.89	0.89	0.89	0.90	0.02
Inertia	Inertia	0.19	0.19	0.19	0.20	0.03	0.21	0.21	0.20	0.22	0.03
Haralick Correlation	HarCor	317.48	375.56	303.63	447.49	242.23	410.36	603.40	467.71	739.10	456.94
Short Run Emphasis	ShortRE	0.33	0.34	0.35	0.34	0.03	0.35	0.35	0.34	0.36	0.03
Long Run Emphasis	LongRE	16.58	16.51	16.01	17.01	1.68	15.44	15.64	15.10	16.18	1.81
Grey Level Non Uniformity	GreyLNU	2405.84	2374.26	2272.67	2475.84	342.08	2240.61	2249.65	2158.63	2340.67	306.50
Run Length Non Uniformity	RunLNU	1239.23	1287.96	1209.65	1366.27	263.72	1443.88	1459.22	1367.19	1551.25	309.91
Low Grey Level Run Emphasis	LowGLRE	0.06	0.06	0.06	0.06	0.01	0.06	0.06	0.05	0.06	0.01
High Grey Level Run Emphasis	HighGLRE	19.10	19.98	18.76	21.19	4.09	21.05	22.47	20.95	23.98	5.11
Short Run Low Grey Level Emphasis	ShortRLowGLE	0.02	0.02	0.02	0.02	0.00	0.02	0.02	0.02	0.02	0.00
Short Run High Grey Level Emphasis	ShortRHighGLE	6.96	7.25	6.72	7.77	1.77	8.15	8.56	7.89	9.24	2.27
Long Run Low Grey Level Emphasis	LongRLowGLE	1.05	1.05	0.98	1.11	0.23	0.95	0.95	0.87	1.03	0.28
Long Run High Grey Level Emphasis	LongRHighGLE	299.09	303.82	283.69	323.95	67.80	309.96	317.43	298.88	335.97	62.45
Bone Volume (%)	BV/TV	0.54	0.54	0.48	0.60	0.20	0.60	0.58	0.52	0.64	0.20
Trabecular Thickness (mm)	Tb.Th	0.35	0.38	0.33	0.43	0.16	0.41	0.44	0.38	0.50	0.19
Trabecular Separation (mm)	Tb.Sp	0.28	0.34	0.27	0.40	0.21	0.26	0.35	0.25	0.44	0.31
Trabecular Number (mm^−1^)	Tb.N	1.47	1.44	1.38	1.51	0.23	1.45	1.36	1.28	1.44	0.28
Bone Surface to Bone Volume Ratio (mm^−1^)	BS/BV	5.79	6.08	5.43	6.73	2.18	4.89	5.30	4.65	5.95	2.18

CI: Confidence Interval; SD: Standard Deviation.

**Table 3:** Descriptive values for each biomolecular variable.

Variables	Abbreviation	Control Group (n = 46) Female (39) Male (7)					TMJ OA Group (n = 46) Female (39) Male (7)				
		Median	Mean	95% CI		SD	Median	Mean	95% CI		SD
Biomolecular Variables (pg/ml)				Lower	Upper				Lower	Upper	
Angiogenin Serum	ANG_Ser	1467.10	1454.86	1389.09	1520.62	221.46	1459.05	1457.15	1368.65	1545.65	298.02
BDNF Serum	BDNF_Ser	280.25	719.56	378.08	1061.04	1149.92	286.35	1121.62	544.88	1698.35	1942.12
CXCL16 Serum	CXCL16_Ser	3726.70	3741.37	3550.00	3932.73	644.40	3827.50	3988.57	3687.27	4289.87	1014.61
ENA-78 Serum	ENA-78_Ser	348.70	664.23	453.24	875.21	710.48	276.40	593.05	394.98	791.11	666.97
MMP3 Serum	MMP3_Ser	2358.25	2373.10	2073.33	2672.87	1009.45	2305.20	2367.03	2091.83	2642.24	926.72
MMP7 Serum	MMP7_Ser	496.55	527.66	444.90	610.42	278.69	453.75	554.15	419.32	688.98	454.02
OPG Serum	OPG_Ser	2539.15	3010.92	2165.32	3856.52	2847.49	2428.10	3116.79	2415.06	3818.51	2363.01
PAI-1 Serum	PAI-1_Ser	6505.60	7930.35	6486.74	9373.96	4861.24	6693.65	7237.11	5904.80	8569.41	4486.42
TGF-β1 Serum	TGF-β1_Ser	91.20	140.68	98.90	182.47	140.70	99.15	177.84	103.81	251.87	249.29
TIMP-1 Serum	TIMP-1_Ser	7382.15	7280.32	7020.93	7539.71	873.47	7351.65	7382.74	7099.45	7666.03	953.95
TRANCE Serum	TRANCE_Ser	2078.70	2200.67	1885.39	2515.95	1061.69	2507.15	2560.51	2231.90	2889.12	1106.56
VE-cadherin Serum	VE-cad_Ser	6259.20	6527.08	5140.64	7913.53	4668.73	5308.05	6154.80	4988.12	7321.48	3928.70
VEGF Serum	VEGF_Ser	93.90	115.32	76.18	154.46	131.80	87.30	117.40	85.32	149.47	108.01
Angiogenin Saliva	ANG_Sal	721.85	720.83	652.98	788.69	228.48	754.05	758.02	702.29	813.74	187.65
BDNF Saliva	BDNF_ Sal	5.20	7.60	5.13	10.08	8.34	3.95	8.32	4.03	12.62	14.47
CXCL16 Saliva	CXCL16_Sal	109.40	183.94	121.59	246.29	209.95	100.60	207.09	130.24	283.95	258.80
ENA-78 Saliva	ENA-78_Sal	2424.60	2218.02	1925.14	2510.90	986.25	2482.60	2410.40	2087.41	2733.39	1087.63
MMP7 Saliva	MMP7_Sal	3290.90	3615.28	2949.23	4281.33	2242.87	3594.20	3666.79	3040.77	4292.82	2108.08
OPG Saliva	OPG_Sal	555.75	855.69	560.07	1151.32	995.49	732.30	1329.53	754.68	1904.38	1935.77
PAI-1 Saliva	PAI-1_Sal	24.40	85.02	45.07	124.96	134.51	40.40	93.14	32.77	153.52	203.32
TGF-β1 Saliva	TGF-β1_Sal	40.70	64.66	41.86	87.46	76.78	54.45	69.20	46.64	91.76	75.97
TIMP-1 Saliva	TIMP-1_Sal	4070.90	3963.81	3663.14	4264.48	1012.49	3889.80	3880.69	3664.71	4096.67	727.30
TRANCE Saliva	TRANCE_Sal	627.60	794.52	533.49	1055.56	879.02	698.75	1041.55	622.45	1460.64	1411.27
VE-cadherin Saliva	VE-cad_Sal	643.10	1008.24	657.65	1358.84	1180.60	666.70	1313.82	585.33	2042.30	2453.11
VEGF Saliva	VEGF_Sal	1181.35	1342.62	1125.23	1560.02	732.07	1441.65	1419.39	1281.99	1556.79	462.68

CI: Confidence Interval; SD: Standard Deviation.

### Radiomic features differentiate control subjects and TMJ OA patients

**Figure 2:**
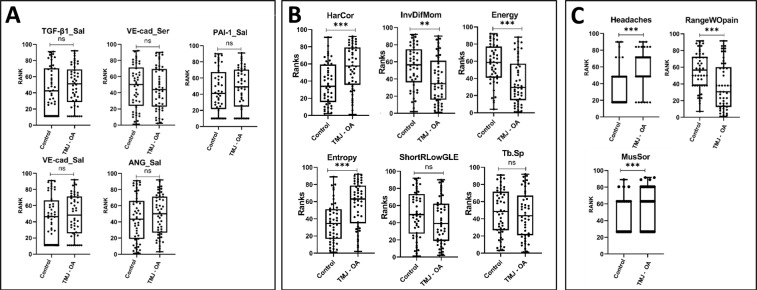
Mann-Whitney U test comparison between the TMJ OA and control groups showing the variables included in our diagnosis prediction models; (**A**) Biomolecular features; (**B**) Radiomics features; (**C**) Clinical features.

We used a non-invasive protocol validated by our group to detect the initial morphological changes in the mandibular condyle trabecular bone based on radiomics information^10^. We extracted 20 imaging features (GLCM, GLRLM and bone morphometry described in Table 2 ^12,38^. These radiomic features were tested using the Mann-Whitney U test (Fig. 2B, Supplementary Fig. 1B and Supplementary Data 1) for group comparisons. From the 20 variables, 13 showed statistically significant differences between the disease and control groups. These findings suggest, and corroborate the literature, that the trabecular bone has an important role in the TMJ OA pathogenesis^6,17,43^.

### Control and TMJ OA patients present similar expression levels of selected serum and saliva protein biomarkers

We selected proteins that have previously been detected in the TMJ synovial fluid of OA patients^39^. We collected saliva and serum using less invasive procedures and promising screening diagnostic tools^44^ to evaluate the diagnostic performance of each protein and their interactions with radiomics and clinical markers. To analyze each protein’s expression level, we used a customized human protein micro-array kit (RayBiotech, Norcross, GA) with duplicate samples for each patient, controlling the standard curve and limiting expression as can be seen in Supplementary Fig. 2. In both the saliva and serum samples, the levels of proteins did not differ, and large data distribution variability was observed, as described in Table 3. We show in Fig. 2A and Supplementary Data 2, the Mann-Whitney U-test results for comparison between the TMJ OA and control groups. Even though our results showed no differences between our groups, the next sections detail the contribution and diagnostic performance of those proteins to diagnose TMJ OA status

### Clinical features differentiate control subjects and TMJ OA patients

We present the Mann-Whitney U test in Supplementary Fig. 1c and Supplementary Data 3 for comparison of both groups for the following clinical variables: RangeAssMax, MusSor, RangeWOpain, Headaches and RangeUnaMax, defined in Table 1. We chose these features because they were measured in both groups and are part of the “*Diagnostic Criteria for Temporomandibular Disorders (DC/TMD) for Clinical and Research Applications: Recommendations of the International RDC/TMD Consortium Network and Orofacial Pain Special Interest Group*”^15^. Our results show that only RangeAssMax and RangeUnaMax were not statistically significantly different between the TMJ OA and control groups. The clinical features that presented statistically significant differences were correlated with pain or limited by pain. For example, for the maximum opening without pain (RangeWOpain), patients were instructed to open their mouths until they start to feel pain within their TMJs. This approach reduces the amount of opening for the TMJ OA patients that often present pain during opening; however, when the patients were asked to open the mouth as much as they could even with pain (i.e., RangeUnaMAx) the values were not statistically significant between the groups. In Fig. 2C, we display only the variables that were included in our diagnostic models, described in the following sections.

### Diagnostic performance to predict TMJ OA status

**Figure 3:**
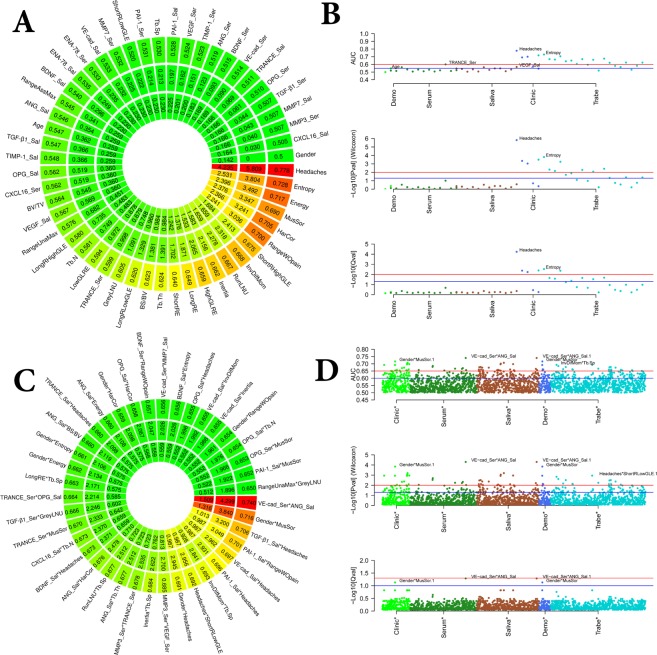
(**A,C**) General association analysis of risk factors. The outer circle shows the AUC, middle circle shows the p-values, and the inner circle shows the q-values for each single feature. (**A,C**) for 52 features, and 39 interactions, respectively. (**B,D**) for 52 features and 1326 interactions, respectively. (**B,D**) The upper graphic shows the AUC, the middle graph shows the p-values, and the lower category shows the q-values for each category of features.

**Figure 4:**
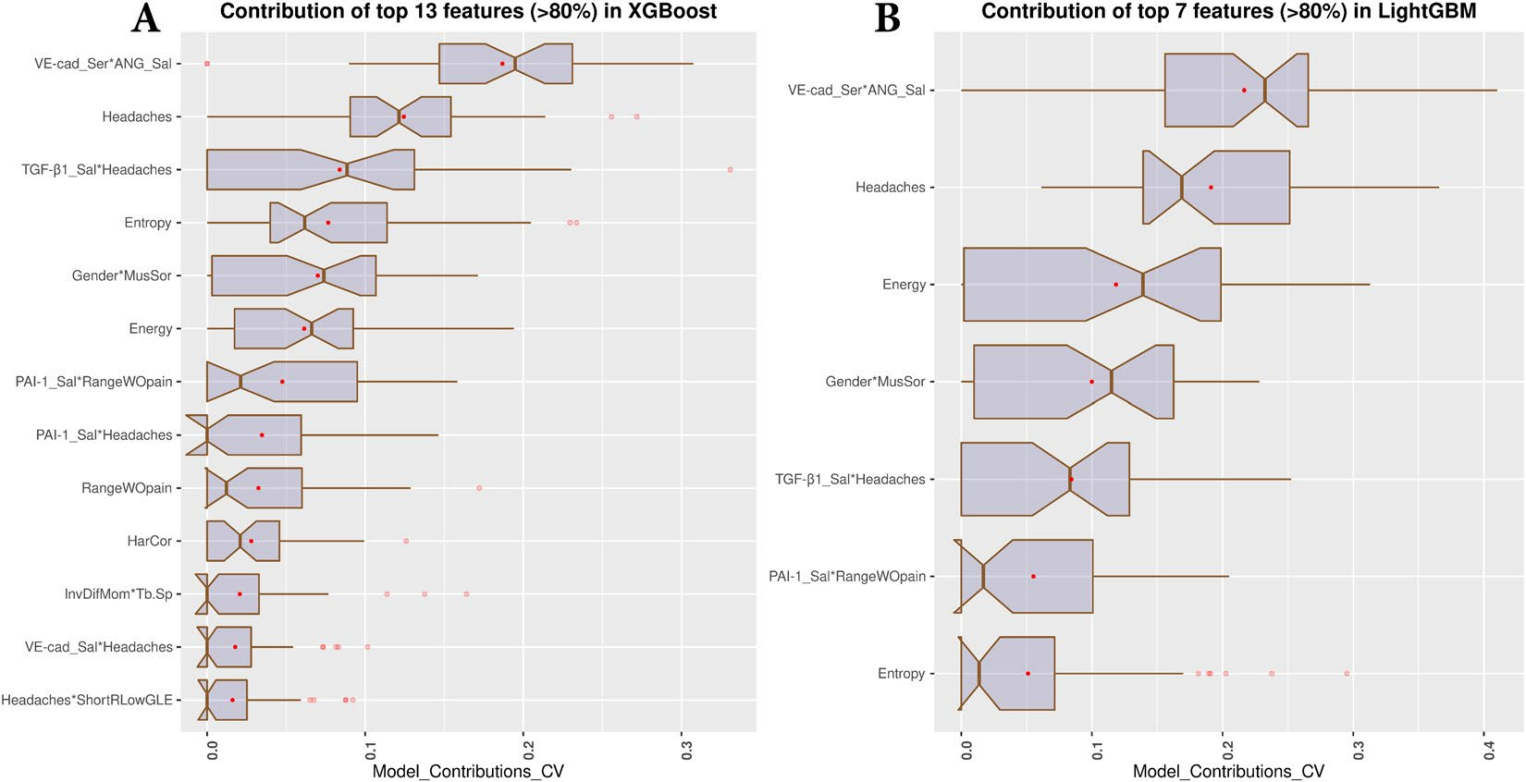
Top features with mean contribution (according to feature importance) greater than 80% for 10 times 5-fold CV. (**A**) Top 13 features in the XGBoost prediction model for 10 times 5-fold CV; (**B**) Top 7 features the LightGBM prediction model for 10 times 5-fold CV.

We had 55 features shown in Tables 1–3 plus gender; however, 4 clinical features are expressed only in OA patients, and for this reason, were not included in the next analysis, resulting in a total of 52. In Fig. 3A, we show the values for the Area Under the curve (AUC), p-value, and q-value for 52 features. Figure 3B shows the AUC (upper), p-value (medium) and q-value (lower) for each category of variables (biological, clinical and radiomics). Most of the features with significant AUC values are clinical or radiomics; no biomolecular feature is detected with AUC > 0.65; nevertheless, it is shown that the interaction of biomolecular features can attain an AUC of 0.74 (Fig. 3C) and have a large contribution in prediction of TMJ OA status(Figs. 3B and 4).

### Contributions assessment of top features and interactions

Top features were filtered with {AUC > 0.7} calculated from the training subjects and then fed into an XGBoost^30^/LightGBM^31^ model to make diagnostic predictions. More details can be found in the Method section. We demonstrate (Fig. 4) the contributions of the top (>80% contribution in sum) features selected from the 52 features with mutual interactions, according to feature importance of XGBoost (Fig. 4A) and LightGBM (Fig. 4B) prediction models, using10 times 5-fold cross-validation,. We find that 13 features using the XGBoost model have a mean contribution larger than 80%, while for LightGBM a subset including 7 features contributes >80% information.

### Diagnosis of TMJ OA status based on the top features and interactions

**Figure 5:**
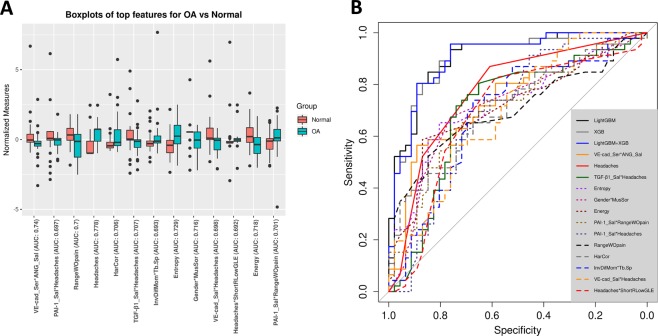
Top features to diagnose disease status. (**A**) Boxplots of normalized features; (**B**) ROC curves of diagnostic sensitivity and specificity for individual features with top mean importance and the mean prediction of XGBoost, LightGBM and their ensemble method IN the 10-times 5-fold CV.

After the selection of the best features and interactions (Fig. 4), Fig. 5A displays the boxplots for comparison between OA and control groups. Figure 5B is showing the ROC curves of diagnostic sensitivity and specificity for individual features with top mean importance and the mean prediction of XGBoost, LightGBM and their ensemble method IN the 10-times 5-fold CV. The accuracy, precision, recall, AUC and F1-score of five described methods (${{\mathscr{F}}}_{1},\,{{\mathscr{P}}}_{1}$), (${{\mathscr{F}}}_{2},\,{{\mathscr{P}}}_{1}$), (${{\mathscr{F}}}_{2},\,{{\mathscr{P}}}_{2}$), (${{\mathscr{F}}}_{2},\,{{\mathscr{P}}}_{3}$), (${{\mathscr{F}}}_{2},\,{{\mathscr{P}}}_{2}$), (${{\mathscr{F}}}_{2},\,{{\mathscr{P}}}_{2}+{{\mathscr{P}}}_{3}$) and (${{\mathscr{F}}}_{2},\,{{\mathscr{P}}}_{4}$) (see method section) are shown in Table 4 using 10 times’ 5-fold cross-validation. Even though the main effect of biomolecular features is low, their interaction effects with clinical and radiomics features are important in diagnosis. For the 10 times’ 5-fold CV, the XGBoost + LightGBM with the interaction features achieves the best average accuracy of 0.823, AUC 0.870, and F1-score 0.823.

**Table 4:** Accuracy, precision, recall, AUROC and F1-score for the methods tested with different hyperparameters evaluated by 10 times 5-fold Cross Validation (mean and standard deviation of the 10 times’ division).

	(η,W,C,S)	Accuracy	Precision.OA	Precision.Control	
(${{\mathscr{F}}}_{1},\,{{\mathscr{P}}}_{1}$)	—	0.737 (0.025)	0.760 (0.032)	0.718 (0.023)	
(${{\mathscr{F}}}_{2},\,{{\mathscr{P}}}_{1}$)	—	0.763 (0.050)	0.770 (0.060)	0.762 (0.053)	
(${{\mathscr{F}}}_{2},\,{{\mathscr{P}}}_{2}$)	(0.001,2,0.7,0.5)	0.793 (0.032)	0.793 (0.028)	0.797 (0.046)	
(${{\mathscr{F}}}_{2},\,{{\mathscr{P}}}_{2}$)	(0.001,1,0.7,0.5)	0.804 (0.022)	0.804 (0.020)	0.808 (0.038)	
(${{\mathscr{F}}}_{2},\,{{\mathscr{P}}}_{2}$)	(0.01, 2, 0.7,0.5)	0.807 (0.034)	0.804 (0.029)	0.812 (0.046)	
(${{\mathscr{F}}}_{2},\,{{\mathscr{P}}}_{2}$)	(0.01, 1, 0.7,0.5)	0.813 (0.023)	0.811 (0.022)	0.817 (0.032)	
(${{\mathscr{F}}}_{2},\,{{\mathscr{P}}}_{2}$)	(0.01,1,0.5,0.5)	0.814 (0.025)	0.807 (0.026)	0.822 (0.028)	
(${{\mathscr{F}}}_{2},\,{{\mathscr{P}}}_{3}$)	(0.01,1,0.7,0.5)	0.802 (0.039)	0796 (0.035])	0.811 (0.054)	
(${{\mathscr{F}}}_{2},\,{{\mathscr{P}}}_{3}$)	(0.001,1,0.7,0.5)	0.800 (0.034)	0.795 (0.033)	0.807 (0.043)	
(${{\mathscr{F}}}_{2},\,{{\mathscr{P}}}_{3}$)	(0.01,2,0.7,0.5)	0.805 (0.044)	0.800 (0.039)	0.814 (0.058)	
(${{\mathscr{F}}}_{2},\,{{\mathscr{P}}}_{3}$)	(0.01,2,0.7,0.7)	0.805 (0.044)	0.800 (0.039)	0.814 (0.058)	
(${{\mathscr{F}}}_{2},\,{{\mathscr{P}}}_{4}$)	—	0.795 (0.035)	0.790 (0.036)	0.802 (0.042)	
(${{\mathscr{F}}}_{2},\,{{\mathscr{P}}}_{2}+{{\mathscr{P}}}_{3}$)	**(0.01,1,0.5,0.5)+ (0.01,2,0.7,0.5)**	**0.823 (0.029)**	**0.815 (0.033)**	**0.833 (0.035)**	
	**(η,W,C,S)**	**Recall.OA**	**Recall.Control**	**AUC**	**Mean.F1.Score**
(${{\mathscr{F}}}_{1},\,{{\mathscr{P}}}_{1}$)	—	0.693 (0.030)	0.780 (0.038)	0.805 (0.026)	0.736 (0.025)
(${{\mathscr{F}}}_{2},\,{{\mathscr{P}}}_{1}$)	—	0.757 (0.072)	0.770 (0.080)	0.838 (0.024)	0.762 (0.051)
(${{\mathscr{F}}}_{2},\,{{\mathscr{P}}}_{2}$)	(0.001,2,0.7,0.5)	0.796 (0.061)	0.791 (0.036)	0.858 (0.025)	0.793 (0.032)
(${{\mathscr{F}}}_{2},\,{{\mathscr{P}}}_{2}$)	(0.001,1,0.7,0.5)	0.807 (0.053)	0.802 (0.030)	0.861 (0.033)	0.804 (0.022)
(${{\mathscr{F}}}_{2},\,{{\mathscr{P}}}_{2}$)	(0.01, 2, 0.7,0.5)	0.811 (0.060)	0.802 (0.347)	0.868 (0.031)	0.806 (0.034)
(${{\mathscr{F}}}_{2},\,{{\mathscr{P}}}_{2}$)	(0.01, 1, 0.7,0.5)	0.817 (0.040)	0.809 (0.027)	0.875 (0.038)	0.813 (0.023)
(${{\mathscr{F}}}_{2},\,{{\mathscr{P}}}_{2}$)	(0.01,1,0.5,0.5)	0.826 (0.031)	0.802 (0.030)	0.870 (0.029)	0.814 (0.025)
(${{\mathscr{F}}}_{2},\,{{\mathscr{P}}}_{3}$)	(0.01,1,0.7,0.5)	0.813 (0.063)	0.791 (0.040)	0.859 (0.035)	0.802 (0.039)
(${{\mathscr{F}}}_{2},\,{{\mathscr{P}}}_{3}$)	(0.001,1,0.7,0.5)	0.809 (0.050)	0.791 (0.039)	0.864 (0.029)	0.800 (0.034)
(${{\mathscr{F}}}_{2},\,{{\mathscr{P}}}_{3}$)	(0.01,2,0.7,0.5)	0.815 (0.068)	0.796 (0.044)	0.861 (0.035)	0.805 (0.044)
(${{\mathscr{F}}}_{2},\,{{\mathscr{P}}}_{3}$)	(0.01,2,0.7,0.7)	0.815 (0.068)	0.796 (0.044)	0.861 (0.035)	0.805 (0.044)
(${{\mathscr{F}}}_{2},\,{{\mathscr{P}}}_{4}$)	—	0.804 (0.048)	0.785 (0.044)	0.795 (0.035)	0.794 (0.035)
(${{\mathscr{F}}}_{2},\,{{\mathscr{P}}}_{2}+{{\mathscr{P}}}_{3}$)	**(0.01,1,0.5,0.5)+** **(0.01,2,0.7,0.5)**	**0.837 (0.044)**	**0.809 (0.043)**	**0.870 (0.033)**	**0.823 (0.029)**

### Cross-validation to control for overfitting

In order to select risk factors from the high-dimensional 52 features plus 1326 interactions, the control of overfitting is necessary. To take advantage of a larger training sample size to fulfill this aim, we use the 10 times’ 5-fold cross-validation and give evaluation and comparison using the average performance of different approaches on validation subjects. Each time in the 10 times’ 5-fold CV, we select hyperparameters, i.e., the iteration steps, by further splitting the training subjects for 10-fold (or 5-fold) cross-validation.

## Discussion

We report here, the diagnostic performance of machine learning approaches to predict TMJ osteoarthritis status. Through data acquisition, management and processing, we achieve one of the main challenges of healthcare delivery, which is to integrate the patient data information from multiple sources for accurate diagnosis and meaningful indicators of individual health^45^. To obtain patient-specific, precise diagnostic information, Data Science has become indispensable in medicine, with the integration of data capture, management/processing and in-depth analysis with rigorous and standardized protocols^46,47^.

**Figure 6:**
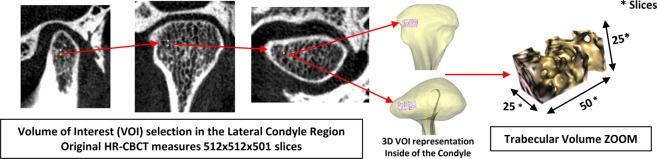
Image volume of interested selection to extract radiomics and bone morphometry features.

In the carefully controlled data acquisition methods of our study, all subjects had the same imaging acquisition protocol, all clinical assessments were performed by the same pain specialist, and a single investigator collected the clinical, biological and imaging data. This study database was composed initially of 107 subjects, and 15 subjects were excluded due to incomplete or inadequate quality of data. As part of the data management and processing, we extracted radiomics information from each subject’s HR-CBCT scan (Fig. 6) to obtain information that is hidden to the clinicians’ naked eyes^48^. Our result in Supplementary Fig. 1 shows that most radiomics features were able to differentiate between control and TMJ OA patients. Advanced statistical learning approaches, shown in Fig. 4, demonstrate among the radiomics features, that Entropy, Energy, HarCor, are included in our most accurate prediction models and corroborate our previous findings that found correlations between these features and the bone status^38^, where a decreased energy was associated with bone sclerosis/loss, and the increased values for HarCor and Entropy was correlated to bone sclerosis/loss. For the knee OA diagnosis, Brahim *et al*.^26^, (2019) using imaging features, evaluated the performance of machine learning algorithms to detect the disease, with x-rays radiographic﻿ from the public dataset Osteoarthritis Initiative (OAI). Their results showed an OA detection with 82.98% of accuracy using Random Forest and Naive Bayes classifiers. Even though the results were good, a standardization of the images was necessary to classify the images from multi-centers correctly. Here, we addressed this challenge, using a rigorous protocol for the imaging markers; however, our sample size (n = 46 per group) in comparison with Brahim *et al*.^26^ (n = 516 per group) was not enough to get a good accuracy only using the radiomics, where we found an AUC of 0.70 approximately (Energy, Entropy, Haralick Correlation, Inverse Difference Moment and Trabecular Separation). For this reason, we also included clinical features and biomolecular information that improved our diagnostic model performance. Also, we have found in the literature only two papers showing the application of machine learning approaches in the temporomandibular joint osteoarthritis diagnosis, and both were focused on developing a model only for bone surface classification^35,36^.

All clinical data were obtained using the DC/TMD^15^ criteria. We have chosen features measured in both groups, as described in Fig. 3, where headaches had the highest AUC among all the features. This marker is highly correlated to temporomandibular disorders in the literature^49^, and now our study shows its improved diagnostic performance to predict OA status in conjunction with other features and machine learning approaches. Interestingly, the interaction of headaches with TGF-β1_Sal together was one of the top features with >80% mean contribution to information gain in our statistical learning models. A possible clinical explanation is that patients with headaches had increased levels of this protein, as previous studies have indicated the expression and correlation of this cytokine with mandibular bone degradation in TMJ OA patients^50–53^. Other clinical markers included in our disease model were: RangeWOpain and its interaction with PAI-1_Sal. As TMJ OA patients present with pain in their TMJs, the decreased amount of mouth opening without pain was an important disease sign with an AUC of 0.70; and its interaction with PAI-1_Sal was an exciting finding, as this feature was increased in the OA patients (Fig. 4). PAI-1 is a serine protease inhibitor of tissue plasminogen activator and prevents the formation of plasmin; in OA, PAI-1 has a role in the cascade of enzymatic activities, compromising repair and increasing the cartilage degradation^19,54^. A recent study by our group showed that PAI-1 is correlated with areas of flattening in the lateral surface of mandibular condyles with OA^39^, corroborating the results of this study. For the demographic’s aspect, Gender and its interaction with MusSor was another significant feature included in our statistical prediction models. However, as TMJ OA prevalence is higher in women^55^, a limitation of this study is the unequal number of male and female subjects; out of the 46 subjects in each group (sample size n = 92), 39 were females and only 7 males. The role of MusSor in this interaction may be due to differences in pain sensitivity in women and men^56^, and to the central sensitization caused by painful osteoarthritis as patients with temporomandibular disorders also presented higher prevalence of muscle-related symptoms^57^. In our study, the variables and/or interactions of: PAI-1_Sal*Headaches (AUC: 0.697), RangeWOpain (AUC: 0.7), Headaches (AUC: 0.778), TGF-β1_Sal*Headaches (AUC: 0.707), Gender*MusSor (AUC: 0.716), VE-cad_Sal*Headaches (AUC: 0.698), Headaches*ShortRLowGLE (AUC: 0.692)), PAI-1_Sal*RangeWOpain (AUC: 0.701) were the clinical features presenting >80% mean contribution to information gain, included in LightGBM and/or XGBoost prediction models. A study from 2013^58^ that also investigated clinical markers (﻿age, sex, weight, height), and genes (FAS844, FAS670, FASL377, FASL124) with support vector machines and probabilistic neural networks found a high classification performance, showing an AUC of approximately 0.95. This study indicates that our findings may be improved with the addition of genetics information. However, besides the challenges involving genetic studies, here, we used relatively simple approaches to assess our features, that could be applied to the clinical practice.

For the biomolecular markers, no differences between OA and control subjects (Supplementary Fig. 2A) were found; however, our prediction models show that the interaction between VE-cad_Ser*ANG_Sal (AUC: 0.74), PAI-1_Sal*Headaches, TGF-β1_Sal*Headaches (AUC: 0.707), VE-cad_Sal*Headaches (AUC: 0.698), PAI-1_Sal*RangeWOpain (AUC: 0.701), TGF-β1_Sal*Headaches and PAI-1_Sal*RangeWOpain (AUC: 0.701) are top features with mean>80% contribution to the information gain in the XGBoost and LightGBM predictive models. As markers of inflammation, VE-cad, ANG, TGF-β1 and PAI-1 have been previously shown^39^ to be expressed in the TMJ synovial fluid and plasma and to be correlated with the condylar morphology in OA patients. It should be highlighted that in the present study, those markers were obtained from saliva and blood samples utilizing less invasive procedures for the patient, and circulating levels of pro-inflammatory proteins have been shown to contribute to the pathophysiology of disorders of the TMJ^59^. In addition, saliva has been described as a promising, accurate and non-invasive tool for a reliable diagnosis^44^. A study from Kellesarian *et al*.^60^, showed the cytokines profile in the synovial fluid of patients with TMJ disorders, and the most proteins that we used in our study were listed in their systematic review. For the knees OA, Hear *et al*.^28^, (2014) also evaluated the performance of machine learning algorithms using artificial neural network (TreeBagger decision tree) and cytokines from serum as features to classify patients with TMJ OA, rheumatoid arthritis and normal. Interestingly, the authors found only 12 statistically significant differences in the cytokines from the 38 studied, and the patients were classified based on late symptoms. The CNN showed the high performance to classify the patients (sensitivity and specify higher than 90%), and the author suggests that ﻿a combination of the cytokines is more critical for the classification than the individual levels, going towards to the findings of our present study.

In a diagnostic perspective, Ahmed *et al*.^61^ aimed to assess the early stages of knee OA. They used ﻿random forest, stepwise generalized linear model, generalized linear models with elastic net as classifiers and proteins from plasma, synovial fluid and serum as features, using leave one out cross-validation and k-fold cross-validation to test the model’s performance. The subjects were classified as early OA, control, early arthritis rheumatoid, and non-rheumatoid arthritis (other inflammatory arthritis). As results, the highest F1 score was for eOA diagnosis (0.78) and the lowest for non-RA (0.36) with GLMNET algorithm. In our study, we had a similar sample size and goal (detection of OA in an early stage), and we found a slightly better F1 score, close to 0.83. A recent study, in 2017^27^, evaluated ﻿machine learning approaches for the identification of new biomarkers for knee osteoarthritis diagnosis. The baseline number of variables for each participant consisted of 186 features, including questionnaires (demographics, anamneses, pain, nutrition, etc.), radiography, magnetic resonance scores, physical/clinical examinations, biomarkers from serum, and urine, etc. The algorithm used was ﻿RGIFE (random forest algorithm), and for testing the model, they selected 10-fold cross-validation. The authors found five good prediction models, including different subsets of features in each. The overall discrimination of knee OA among the patients was considered good with good (AUC between 0.80 and 0.90). Each prediction model presented multi-source biomarkers such as clinical information, imaging-based information, pain, food questionnaires, and molecular markers. These results suggest that the disease has a complexity etiology, corroborating with our findings and confirming that there is a need to investigate the association of clinical, imaging, and proteins to better categorized this complex disease.

Nowadays, the complex, high-dimensional, and biomedical data from multiple sources benefit from data science, computational advances, and machine learning approaches to improve knowledge in terms of diagnosis, disease classification, clustering data, and disease progression prediction^32,62–64^. For osteoarthritis, studies using mathematical algorithms for diagnosis and personalized treatment decisions are increasing^65^. We have previously shown the diagnostic performance to predict the disease status based on the condylar surface morphology and deep learning approaches^35,40^, and now we show an integrative approach based on clinical, imaging radiomics and biomolecular patient-specific data. A limitation of this study is that the cross-sectional study design does not allow assessment of the disease progression and how different disease stages affect the proposed biomarkers. Other studies that assessed early stages of the knee OA using machine learning approaches^33^, used subjective radiologic interpretation of 2D x-rays, rather than the high-resolution 3D images included in the present study. Here, with the use of the radiomics, clinical, and protein information, our predictive model with XGBoost + LightGBM and 1378 features/interactions showed an accuracy of 0.823, AUC 0.870, and F1-score 0.823 to determine disease status. Future studies using the proposed machine learning models and longitudinal data will provide better information on the feature’s behavior and disease progression.

In conclusion, our in-depth statistical learning analysis was based on the integration and interactions of 52 features. We screened the diagnostic performance of each feature (Figs. 3–5) and built our machine learning models based on the most relevant features. Our final prediction model had an accuracy of 0.823 (SD: 0.029) to predict TMJ OA status using LightGBM + XGBoost with 1378 features interactions. Importantly, we show a comprehensive integration of new tools, data acquisition, management, and approaches to improve articular joint health and predict patient-specific TMJ OA status.

## Methods

We followed the *“Strengthening the Reporting of Observational studies in Epidemiology”* (STROBE) guidelines for observational studies^66^. All experiments were performed in accordance with the guidelines and regulations approved by the Institutional Review Board approval (HUM00105204 and HUM00113199) from the University of Michigan and the informed consent was obtained from all participants.

### Study design, setting and participants

After the Institutional Review Board approval (HUM00105204 and HUM00113199) from the University of Michigan, we enrolled patients and subjects from January 2016 to December 2018 that composed our TMJ OA and Control groups, respectively. This cross-sectional study sample was composed of 92 patients, 46 TMJ OA and 46 age and sex-matched control subjects who were selected based on rigorous inclusion criteria. The general health conditions of the participants included: age between 21–70 years old, no history of cancer, no history of jaw joint trauma, no previous surgery in the TMJ or recent jaw joint injections, absence of systemic diseases; no current pregnancy and no congenital bone or cartilage disease. All patients were examined by a single temporomandibular disorders specialist at the Hospital of the University of Michigan (Medicine Oral Surgery Clinic) through the Diagnostic Criteria for Temporomandibular Disorders (DC/TMD)^15^ for TMJ osteoarthritis diagnosis. The patients were diagnosed as early stages of TMJ osteoarthritis when they presented: pain in at least one TMJ for less than 10 years, TMJ noise during movement or function in the last 30 days and crepitus detected during mandibular excursive movements. The Control group subjects were recruited by advertisement and evaluated for the absence of TMJ OA clinical and radiographic signs and symptoms. The diagnosis for the TMJ OA group and side of choice (left or right) was confirmed utilizing the radiographic criteria^16^, including initial stages of subchondral cyst, erosion, generalized sclerosis and/or osteophytes. For the matching control condyle, the side of choice was the one without any clinical or radiographic findings. The exclusion criteria for the TMJ OA group were patients with middle to chronic TMJ OA diagnosis, evaluated when they present more than 10 years of TMJ pain diagnosis and/or severe stages of bone destruction, subchondral cyst, erosion and generalized sclerosis evaluated using the hr-CBCT by a radiologist.

### Variables

Our study was composed by 3 main sub-groups of variables, which were: biomolecular features (composed by proteins of serum and saliva), imaging features (composed by trabecular bone radiomics and morphometry) and clinical features.

### Biomolecular data

We evaluated 14 proteins in serum and saliva associated with arthritis initiation and progression, such as nociception, inflammation, angiogenesis and bone resorption, which were: 6ckine, Angiogenin, BDNF, CXCL16, ENA-78, MMP-3, MMP-7, OPG, PAI-1, TGFb1, TIMP-1, TRANCE, VE-Cadherin and VEGF. However, the expression of 6ckine was not expressed in the serum and saliva samples in this study, and MMP-3 was not expressed in saliva. The raw data can be seen in the Supplementary Fig. 2. The reason to select those proteins, besides their participation in the TMJ OA inflammation process^60^, was due to our previous studies that detected these markers in the TMJ synovial fluid and saliva of OA patients, showing correlations with bone surface changes^35,39^.

### Blood and saliva acquisition protocol

The participants had 5 ml of venous blood collected by a trained nurse at the University of Michigan. The blood was centrifuged for 20 minutes at 1000 RPM to separate only the serum that was then aliquoted in 2 ml Eppendorf tubes and stored at −80C. For the saliva collection, the participants received a 14 ml sterile test tube with a funnel inserted; they were instructed to tilt their head forward and drip the saliva off into the tube until 2 ml was collected. They were informed to not spit, talk, or swallow during this process^67^.

### Custom micro-array

Custom human quantibody protein microarrays obtained from RayBiotech, Inc. Norcross, GA, was used to quantitatively assess the saliva and serum samples for the 14 specific biomarkers. Each participant had duplicates run for the saliva and serum samples (detailed description provided by Jiang *et al*.^68^ and Huang *et al*.^69^). Supplementary Figures 2, 3 shows the raw values obtained for each participant and the standard curves for each protein.

### Clinical signs and symptoms acquisition protocol

The same investigator collected and measured the clinical signs and symptoms of the participants based on the DC/TMD^15^ criteria. The variables measured and selected for further statistical analysis were: Age pain began in years - TMJ OA Group only, Current Facial Pain -TMJ OA Group only, Worst Facial Pain in last 6 months -TMJ OA Group only, Average Pain -TMJ OA Group only, Last 6 Months Distressed by Headaches, Last 6 Months Distressed by Muscle Soreness, Vertical Range Unassisted Without Pain (mm), Vertical Range Unassisted Maximum (mm), Vertical Range Assisted Maximum (mm).

### Imaging data acquisition

We acquired cone-beam computed tomography scans of each subject using the 3D Accuitomo (J. Morita MFG. CORP Tokyo, Japan) machine at the University of Michigan, School of Dentistry. The protocol for the temporomandibular joint high-resolution CBCT was field of view 40 × 40 mm; 90 kVp, 5 mAs, scanning time of 30.8 s and a voxel size of 0.08 mm^3^. The images were exported in DICOM (.dcm) using the manufacture software: i-Dixel (J. Morita MFG. CORP Tokyo, Japan) and optimization manufacture filter: G_103 + H_009. Finally, the images were coded and de-identified to avoid investigator bias in the statistical analysis.

### Imaging trabecular texture-based features

We previously described the optimal parameters to extract radiomics features from the HR-CBCT scans in our study conditions and we followed these parameters to extract the information from our imaging data, using the BoneTexture module^38^. The region analyzed was the internal condylar lateral region (Fig. 6) due to our pilot results that showed this region to be the most significantly different between Control and TMJ OA patients. The textural information evaluated were: Energy, Entropy, Inverse Difference Moment, Inertia, Haralick Correlation, Short Run Emphasis, Long Run Emphasis, Grey Level Non Uniformity, Run Length Non Uniformity, Low Grey Level Run Emphasis, High Grey Level Run Emphasis, Short Run Low Grey Level Emphasis, Short Run High Grey Level Emphasis, Long Run Low Grey Level Emphasis, Long Run High Grey Level Emphasis, Bone Volume, Trabecular Thickness, Trabecular Separation, Trabecular Number and Bone Surface to Bone Volume Ratio.

### Exploratory tests

We first did a traditional statistical analysis to explore our data and to test the hypothesis that there is no difference between our groups. Our data does not show normality distribution and for this reason, we chose non-parametrical tests for our analysis. The descriptive analysis, Mann-Whitney U test was done using the software GraphPad Prisma V 8.11 (GraphPad Software, Inc., San Diego, CA). For the descriptive analysis, we showed the median in addition to the mean, the 95% confidence intervals and the standard deviation. The Mann-Whitney U test was used to test our hypothesis and we used a two-tailed test with α of 5%.

### Machine learning approaches

We diagnose the OA/control disease status based on the 52 features including five clinical variables, 20 radiomics features, 25 biomolecular features (13 from serum and 12 from saliva) and two demographic variables (age and gender). First, we normalized all features to have zero mean and one standard deviation. Next, we calculated the AUROC (Area under the Receiver Operating Characteristic curve), p-value and q-value^70^ from a two-sample Mann-Whitney U test to evaluate the significance of each feature (Fig. 3). Afterward, we compared four different prediction methods, each of which follows the four steps: (I) Cross-validation to avoid overfitting (II) feature selection (III) risk prediction (IV) method evaluation. We used one-sided paired DeLong test^71,72^ to validate the significance of AUC comparison between different approaches.

### Cross-validation (CV)

We applied the 10 times’ 5-fold CV by taking 4 folds as training and the remaining one-fold as validation with 10 times’ repetition. At each time, we normalized the original 52 features denoted as F1 based on the training subjects and then took the product between each pair of them to generate additional 1326 interactions and denoted the set of 1378 features as ${{\mathscr{F}}}_{2}$. We performed the following two-step procedures by using only the training dataset and feature pools ${{\mathscr{F}}}_{1}$ and ${{\mathscr{F}}}_{2}$, respectively, where ${{\mathscr{F}}}_{1}$ represents the set of original 52 features, and we took the product between each pair of ${{\mathscr{F}}}_{1}$ to generate an additional 1326 interactions and denoted the set of 1378 features as ${{\mathscr{F}}}_{2}$. Afterwards, we applied the 10 times’ 5-fold CV by taking 4 folds as training and the remaining one-fold as validation with 10 times’ repetition. This will further evaluate the sensitivity of the model.

### Feature selection

We calculate the AUC for each single feature in ${{\mathscr{F}}}_{2}$ and select top features according to {*f* ∈ F_1_|AUC of *f* > 0.7} and {*f* ∈ F_2_|AUC of *f* > 0.7} for feature pools met F_1_ and F_2_, respectively.

### Evaluation and risk prediction

We trained the logistic regression model (method P1), Extreme Gradient Boosting (XGBoost; method ${{\mathscr{P}}}_{2}$)^30^, Light Gradient Boosting Machine (LightGBM; method ${{\mathscr{P}}}_{3}$)^31^, and Random Forest (method ${{\mathscr{P}}}_{4}$)^73^ model by using the extracted features from the last step for risk prediction of the validation subject. For both XGBoost and LightGBM models, we fix the depth D = 1, and tune the iteration steps by further splitting the training subjects into training and validation subjects for 10-fold cross validation, where AUC is chosen as the evaluation criterion. We evaluate the prediction performance of six pairs of feature set and methods (${{\mathscr{F}}}_{1},\,{{\mathscr{P}}}_{1}$), (${{\mathscr{F}}}_{2},\,{{\mathscr{P}}}_{1}$), (${{\mathscr{F}}}_{2},\,{{\mathscr{P}}}_{2}$), (${{\mathscr{F}}}_{2},\,{{\mathscr{P}}}_{3}$), (${{\mathscr{F}}}_{2},\,{{\mathscr{P}}}_{4}$) and (${{\mathscr{F}}}_{2},\,{{\mathscr{P}}}_{2}+{{\mathscr{P}}}_{3}$) by using the accuracy, precision, recall, AUROC and ${{\mathscr{F}}}_{1}$-score^74^ on the 10 times 5-fold validation subjects. We also compare the results with other different hyperparameters. For example, we show in Table 4 the results for min_child_weight W∈{1,2}, colsample_bytree C∈{0.5,0.7}, subsample S∈ {0.5,0.7} and the learning rate η∈{0.001,0.01}. Our results showed that the XGBoost and LightGBM model by averaging the prediction probability (${{\mathscr{F}}}_{2},\,{{\mathscr{P}}}_{2}+{{\mathscr{P}}}_{3}$) has the best performance on the validation subjects in the 10 times 5-fold CV; here the combination of XGBoost and LightGBM is recommended for its robustness in 10 times’ 5-fold CV.

## Supplementary information

Supplementary information.

Supplementary information2.

Supplementary information3.

Supplementary information4.

Supplementary information5.

## Data Availability

The data analyzed are available from the corresponding author on a reasonable request.

## Code availability

Source code for the Computation and Integration web system (DSCI) is available at https://github.com/DCBIA-OrthoLab/shiny-tooth, for the DataBase interactor 3D-slicer Plugin: https://github.com/DCBIA-OrthoLab/DatabaseInteractorExtension and for the imaging markers extraction the code is available at https://github.com/Kitware/BoneTextureExtension.
