# Supplementary material for: Osteoarthritis of the Temporomandibular Joint can be diagnosed earlier using biomarkers and machine learning
Source: Sci Rep. 2020 May 15;10:8012. doi: 10.1038/s41598-020-64942-0 (PMC7228972; doi:10.1038/s41598-020-64942-0)
Supplement: Supplementary file 1 — Supplementary information. [file 41598_2020_64942_MOESM1_ESM.docx]

**Description of Supplementary Files**

**Title of the manuscript:** Osteoarthritis of the Temporomandibular Joint can be diagnosed earlier using biomarkers and machine learning.

# Authors: Jonas Bianchi*^1,2^ Antonio Carlos de Oliveira Ruellas^1^, João Roberto Gonçalves^2^, Beatriz Paniagua^3^, Juan Carlos Prieto^4^, Martin Styner^4^, Tengfei Li^5^, Hongtu Zhu^5^, James Sugai^6^, William Giannobile^6^, Erika Benavides^6^, Fabiana Soki^6^, Marilia Yatabe^1^, Lawrence Ashman^7^, David Walker^8^, Reza Soroushmehr^9^, Kayvan Najarian^9^, and Lucia Helena Soares Cevidanes^1^.

**Authors affiliation:**

**^1^**University of Michigan, Department of Orthodontics and Pediatric Dentistry, School of Dentistry, Ann Arbor, MI, 48109, USA. **^2^**São Paulo State University, Department of Pediatric Dentistry, School of Dentistry, Araraquara, SP, 14801-385, Brazil. **^3^**Kitware, Inc., Carrboro, NC, 27510, USA. **^4^**University of North Carolina, Department of Psychiatry and Computer Science, Chapel Hill, NC, 27516, USA. **^5^**University of North Carolina, Department of Biostatistics, Chapel Hill, NC, 27516, USA. **^6^**University of Michigan, Department of Periodontics and Oral Medicine, School of Dentistry, Ann Arbor, MI, 48109, USA. **^7^**University of Michigan, Department of Oral and Maxillofacial Surgery and Hospital Dentistry, School of Dentistry, Department of Biomedical Engineering, and the Biointerfaces Institute Ann Arbor, MI, 48109, USA. **^8^**University of North Carolina, Department of Orthodontics, Chapel Hill, NC, 27516, USA. **^9^**University of Michigan, Center for Integrative Research in Critical Care and Michigan Institute for Data Science, Department of Computational Medicine and Bioinformatics, Ann Arbor, MI, 48109, USA.

***Correspondent author:** bianchij@umich.edu

**DESCRIPTION OF SUPPLEMENTARY FILES:**

**FILE NAME: SUPPLEMENTARY DATA 1**

Description: Mann–Whitney U test and Normality statistical values for the imaging markers.

**FILE NAME: SUPPLEMENTARY DATA 2**

Description: Mann–Whitney U test and Normality statistical values for protein markers.

**FILE NAME: SUPPLEMENTARY DATA 3**

Description: Mann–Whitney U test and Normality statistical values for clinical markers.

**SUPPLEMENTARY FIGURE 1 –** Mann-Whitney U test for each biomarker. A- Proteins comparison using Mann–Whitney U test; B- Imaging comparison using Mann–Whitney U test; C- Clinical comparison using Mann–Whitney U test.

**SUPPLEMENTARY FIGURE 2-** A- Summary report for each protein in serum and saliva and LOD: limit of detection; B. Values in a graph bar for each subject, saliva and serum separately.

**SUPPLEMENTARY FIGURE 3-** Micro-array standard curves for each protein.
