# Supplementary material for: Osteoarthritis of the Temporomandibular Joint can be diagnosed earlier using biomarkers and machine learning
Source: Sci Rep. 2020 May 15;10:8012. doi: 10.1038/s41598-020-64942-0 (PMC7228972; doi:10.1038/s41598-020-64942-0)
Supplement: Supplementary file 2 — Supplementary information2. [file 41598_2020_64942_MOESM2_ESM.pdf]

Supplemental materials: 3 figures.

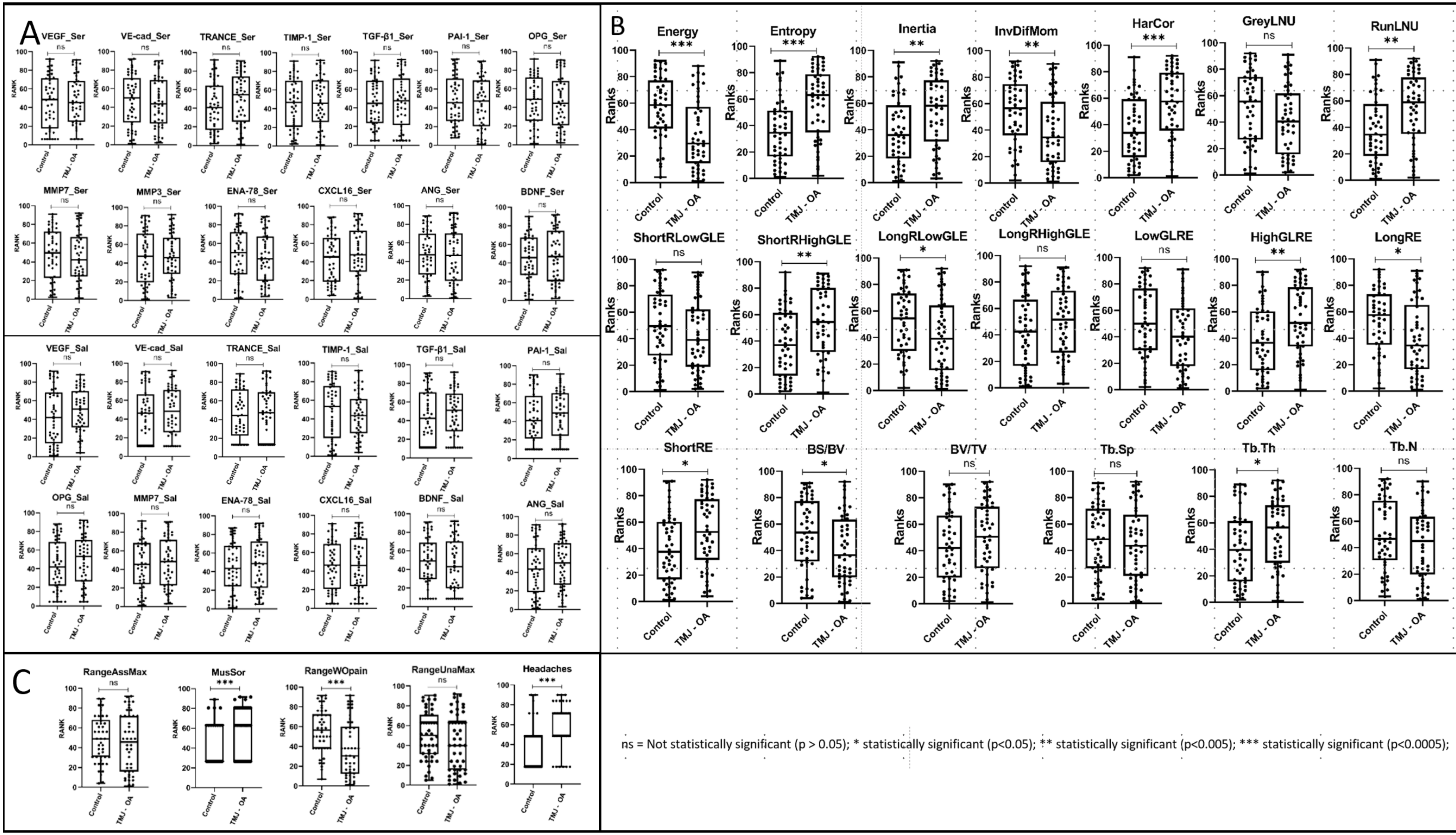

Supplementary Figure 1 – Mann-Whitney U test for each biomarker. A- Proteins comparison using Mann–Whitney U test; B- Imaging comparison using Mann–Whitney U test; C- Clinical comparison using Mann–Whitney U test.

A

Target	LOD (pg/ml)	Best Confident Range	Maximum Value	Highest Reported Value	Lowest Reported Value	*% Below LOD*	% Above LOD but <3xLOD	% in Best Confidence	% Above Maximum
6ckine	4716.2	14,148.7 - 13,333	40,000	16,426.4	0.0	98.8	*1.0*	*0.3*	*0.0*
Angiogenin	1.6	4.9 - 667	2,000	1,310.9	0.2	*0.3*	*0.0*	99.8	*0.0*
BDNF	14.2	42.6 - 3,333	10,000	4,638.9	0.0	42.0	15.8	42.3	*0.0*
CXCL16	12.6	37.9 - 3,333	10,000	4,497.6	0.0	13.8	*4.5*	81.8	*0.0*
ENA-78	19.6	58.7 - 3,333	10,000	5,139.9	0.0	*3.3*	8.5	88.3	*0.0*
MMP-3	16.9	50.8 - 10,000	30,000	3,081.2	0.0	37.5	7.0	55.5	*0.0*
MMP-7	9.9	29.7 - 6,667	20,000	12,776.4	0.0	*2.3*	*0.8*	97.0	*0.0*
OPG	124.2	372.5 - 6,667	20,000	10,509.1	0.0	11.5	7.5	81.0	*0.0*
PAI-1	75.0	225.1 - 13,333	40,000	13,013.2	0.0	34.0	10.8	55.3	*0.0*
TGFb1	125.3	375.8 - 6,667	20,000	801.5	0.0	79.8	19.0	*1.3*	*0.0*
TIMP-1	17.6	52.9 - 6,667	20,000	6,263.3	21.8	*0.0*	*0.3*	99.8	*0.0*
TRANCE	219.1	657.3 - 33,333	100,000	7,032.7	0.0	22.5	13.3	64.3	*0.0*
VE-Cadherin	2297.8	6,893.4 - 66,667	200,000	18,249.7	0.0	62.8	33.8	*3.5*	*0.0*
VEGF	43.6	130.9 - 3,333	10,000	3,137.1	0.0	25.3	21.5	53.3	*0.0*

B

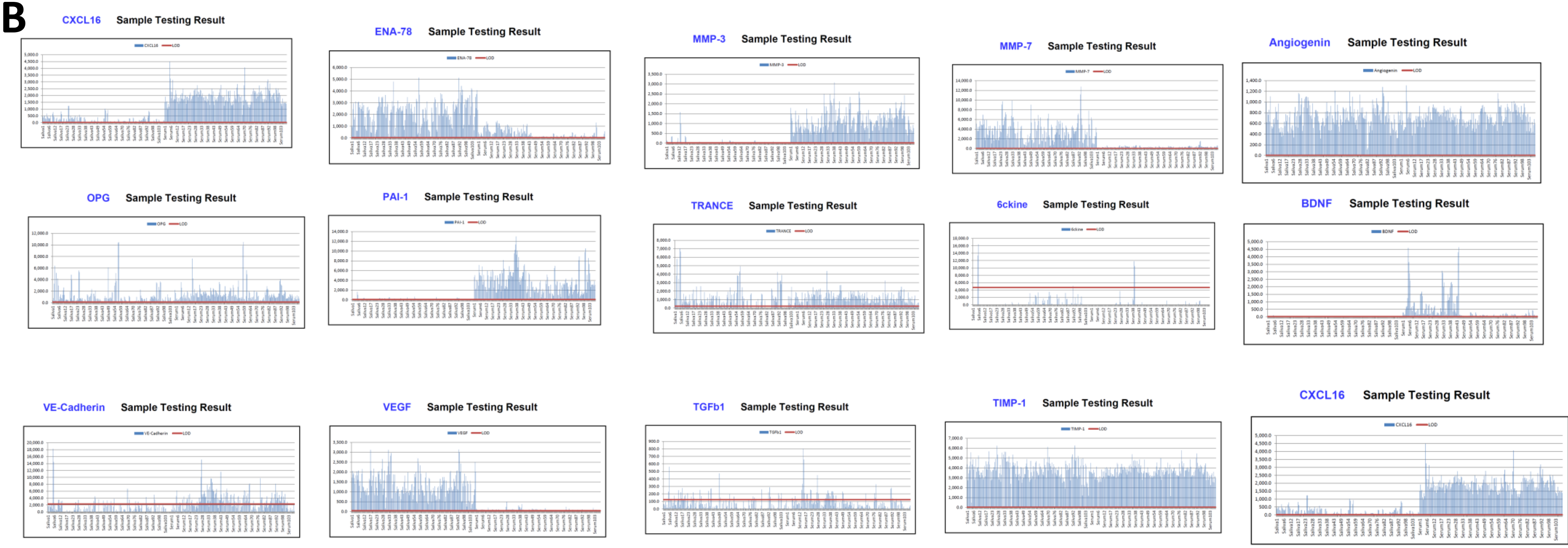

Supplementary Figure 2.A- Summary report for each protein in serum and saliva and LOD: limit of detection; B. Values in a graph bar for each subject, saliva and serum separately.

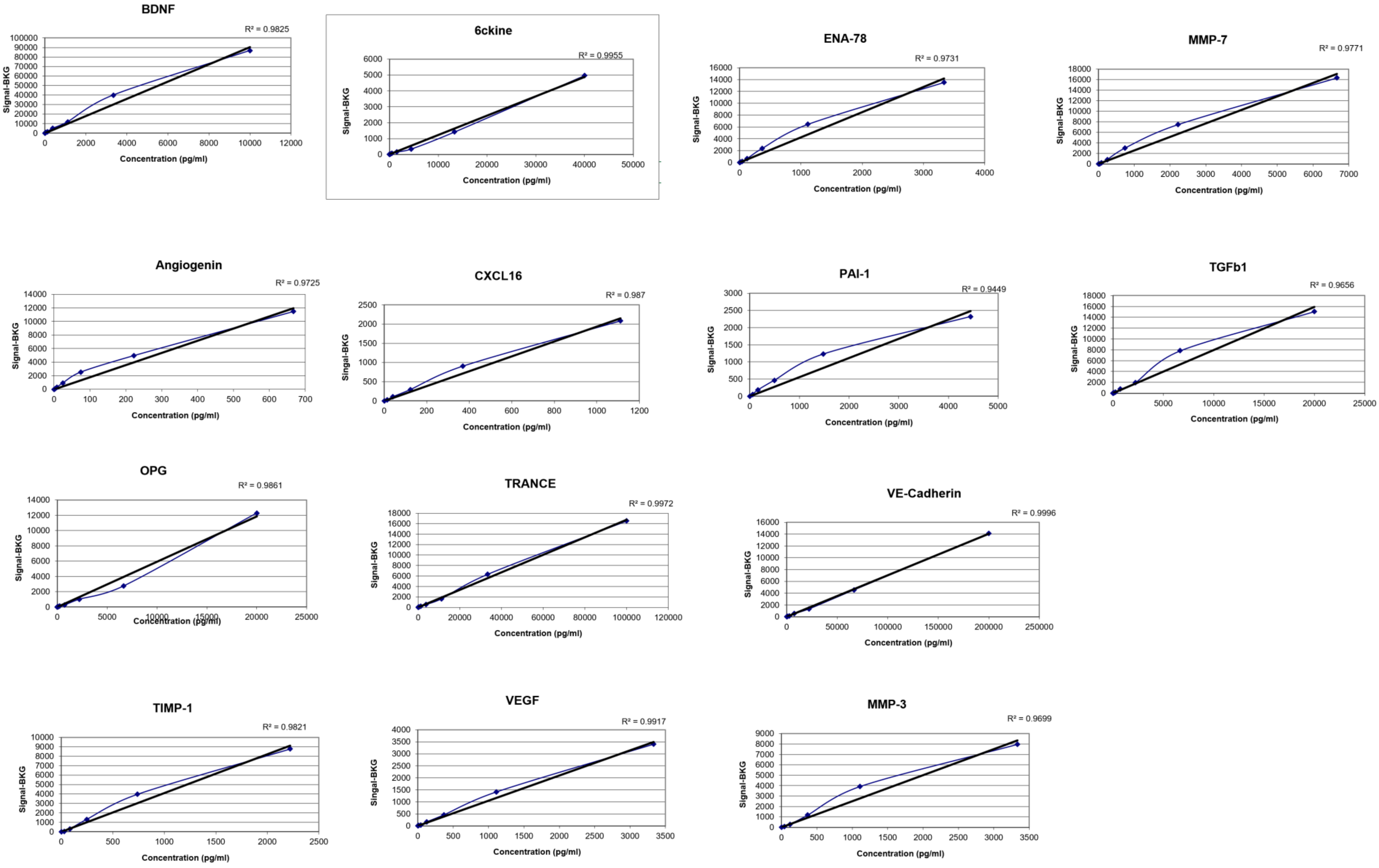

Supplementary Figure 3. Micro-array standard curves for each protein
